# 2-[2-(4-Bromo­phen­yl)hydrazinyl­idene]-1,3-diphenyl­propane-1,3-dione

**DOI:** 10.1107/S1600536811017557

**Published:** 2011-05-14

**Authors:** Carlos Bustos, Luis Alvarez-Thon, Juan-Guillermo Cárcamo, Maria Teresa Garland, Christian Sánchez

**Affiliations:** a Instituto de Ciencias Químicas, Universidad Austral de Chile, Avda. Los Robles s/n, Campus Isla Teja, Casilla 567, Valdivia, Chile; b Departamento de Ciencias Físicas, Universidad Andres Bello, Avda. República 220, Santiago de Chile, Chile; c Instituto de Ciencias Moleculares y Microbiología, Universidad Austral de Chile, Avda. Los Robles s/n, Campus Isla Teja, Casilla 567, Valdivia, Chile; d Laboratorio de Cristalografía, Departamento de Física, Facultad de Ciencias Físicas y Matemáticas, Universidad de Chile, Santiago de Chile, Chile

## Abstract

The conformation of the title mol­ecule, C_21_H_15_BrN_2_O_2_, is stabilized by a weak intra­molecular C—H⋯N hydrogen bond and a strong resonance-assisted N—H⋯O intra­molecular hydrogen bond. In the crystal, the mol­ecules are linked by weak inter­molecular C—H⋯O inter­actions, forming zigzag chains along the *b* axis.

## Related literature

For resonance-assisted hydrogen bonds and related structures, see: Bertolasi *et al.* (1994 ▶). For details of the synthesis, see: Bustos *et al.* (2007 ▶, 2009 ▶); Yao (1964 ▶).
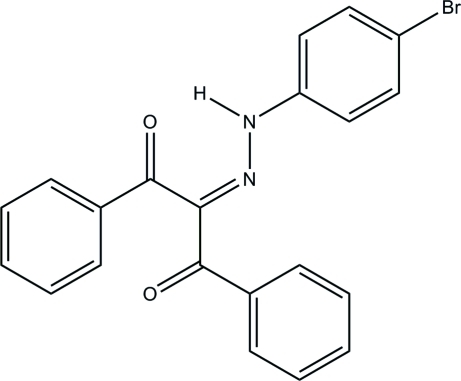


## Experimental

### 

#### Crystal data

C_21_H_15_BrN_2_O_2_
*M* *_r_* = 407.25
Monoclinic, 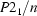

*a* = 12.0273 (9) Å
*b* = 10.2977 (8) Å
*c* = 14.2626 (11) Å
β = 96.452 (1)°
*V* = 1755.3 (2) Å^3^
*Z* = 4
Mo *K*α radiation
μ = 2.36 mm^−1^
*T* = 150 K
0.44 × 0.41 × 0.12 mm

#### Data collection

Bruker D8 Discover diffractometer with SMART CCD area detector
Absorption correction: multi-scan (*SADABS*; Bruker, 2000 ▶) *T*_min_ = 0.368, *T*_max_ = 0.753
13742 measured reflections
3575 independent reflections
3107 reflections with *I* > 2σ(*I*)
*R*_int_ = 0.021

#### Refinement

*R*[*F* ^2^ > 2σ(*F* ^2^)] = 0.030
*wR*(*F* ^2^) = 0.079
*S* = 1.06
3575 reflections
235 parameters
H-atom parameters constrained
Δρ_max_ = 0.67 e Å^−3^
Δρ_min_ = −0.17 e Å^−3^

### 

Data collection: *SMART* (Bruker, 2001 ▶); cell refinement: *SAINT* (Bruker, 2000 ▶); data reduction: *SAINT*; program(s) used to solve structure: *SHELXS97* (Sheldrick, 2008 ▶); program(s) used to refine structure: *SHELXL97* (Sheldrick, 2008 ▶); molecular graphics: *XP* in *SHELXTL-PC* (Sheldrick, 2008 ▶); software used to prepare material for publication: *PLATON* (Spek, 2009 ▶) and *Mercury* (Macrae *et al.*, 2006 ▶).

## Supplementary Material

Crystal structure: contains datablocks global, I. DOI: 10.1107/S1600536811017557/pv2416sup1.cif

Structure factors: contains datablocks I. DOI: 10.1107/S1600536811017557/pv2416Isup2.hkl

Supplementary material file. DOI: 10.1107/S1600536811017557/pv2416Isup3.cml

Additional supplementary materials:  crystallographic information; 3D view; checkCIF report

## Figures and Tables

**Table 1:** Hydrogen-bond geometry (Å, °)

*D*—H⋯*A*	*D*—H	H⋯*A*	*D*⋯*A*	*D*—H⋯*A*
N2—H21⋯O2	0.88	1.90	2.592 (2)	135
C8—H8⋯N1	0.95	2.60	3.060 (3)	110
C17—H17⋯O2^i^	0.95	2.46	3.382 (3)	162

Symmetry code: (i) 
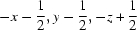
.

**Fig. 1.**
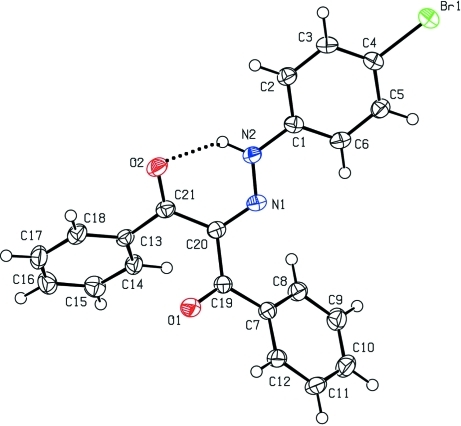
A view of the title compound with the atom numbering scheme. Displacement ellipsoids are drawn at the 50% probability level. The strong intramolecular hydrogen bond (N2–H21···O2) is depicted with dashed lines.

**Fig. 2.**
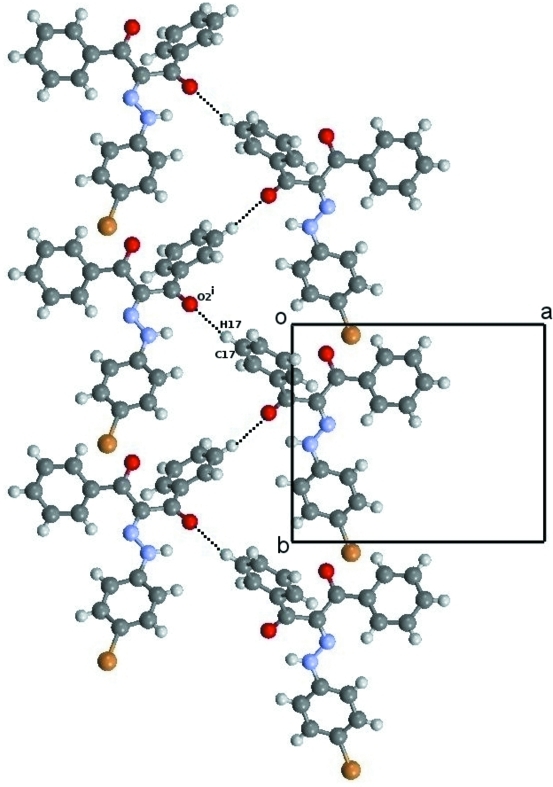
A partial view of the unit cell along the c-axis, showing the formation of zigzag chains of molecules along the b axis.

## supplementary crystallographic information

### Comment

In recent years, much attention has been devoted to structural studies on heterodienic systems forming strong intramolecular hydrogen bonds, N—H···O, assisted by resonance (RAHB, Resonance Assisted Hydrogen Bond) which, *inter alia*, could have potential technological applications as bistate molecular switches (Bertolasi *et al.*, 1994; Bustos *et al.*, 2007). On the other hand, it is well known that the phenyl diazonium salts are capable of coupling with a series of β-diketonate anions to give β-diketohidrazones that contain the N—H···O core (Yao, 1964; Bustos *et al.*, 2007; Bustos *et al.*, 2009). Using this reaction (Yao, 1964) we have prepared the title compound and, in this report, we present its crystal and molecular structure determined by X-ray diffraction method.

The molecular structure of the title compound exhibits a strong intramolecular hydrogen bond (N2–H21···O2) and a weak intramolecular hydrogen bond (C8–H8···N1) (Fig. 1 and Tab. 1). The molecules are linked by weak intermolecular C17–H17···O2^i^ interactions forming zigzag chains along the *b* axis (Fig. 2).

### Experimental

In a 500 ml flask, 1,3-diphenylpropane-1,3-dione (2.24 g, 0.01 mole) was dissolved in an ethanol solution (100 ml) containing of sodium hydroxide (0.4 g, 0.01 mole) and  of sodium acetate (3.65 g, 0.045 mole). The resulting β-diketonate solution was diluted with water to a final volume of about 220 ml, stirred and cooled at 268 K. In another 50 ml beaker a diazonium ion solution was prepared by adding 4-bromoaniline (97%) (1.77 g, 0.01 mole) in 8 ml of hydrochloric acid (5 mol/*L*), cooling at 268 K, and adding a saturated aqueous solution containing sodium nitrite (0.69 g, 0.01 mole). The diazonium salt solution was then added dropwise, with vigorous stirring, into the β-diketonate solution. During the addition a yellow solid precipitate of the title compound was formed which was filtered by suction and washed with an abundant quantity of water (Yield: 96% of crude product). Single crystals suitable for X-ray studies were obtained by recrystallization from a concentrated solution of the compound in ethanol.

### Refinement

The H atoms were placed in geometrically idealized positions and constrained to ride on their parent atoms, with N—H = 0.88 and C—H = 0.95 Å and *U*_iso_(H) = 1.2*U*_eq_(C/N).

### Crystal data

**Table:**

C_21_H_15_BrN_2_O_2_	*F*(000) = 824
*M**_r_* = 407.25	*D*_x_ = 1.541 Mg m^−^^3^
Monoclinic, *P*2_1_/*n*	Mo *K*α radiation, λ = 0.71073 Å
Hall symbol: -P 2yn	Cell parameters from 999 reflections
*a* = 12.0273 (9) Å	θ = 2.1–26.4°
*b* = 10.2977 (8) Å	µ = 2.36 mm^−^^1^
*c* = 14.2626 (11) Å	*T* = 150 K
β = 96.452 (1)°	Polyhedron, yellow
*V* = 1755.3 (2) Å^3^	0.44 × 0.41 × 0.12 mm
*Z* = 4	

### Data collection

**Table:**

Bruker D8 Discover diffractometer with SMART CCD area detector	3575 independent reflections
Radiation source: fine-focus sealed tube	3107 reflections with *I* > 2σ(*I*)
graphite	*R*_int_ = 0.021
φ and ω scans	θ_max_ = 26.4°, θ_min_ = 2.1°
Absorption correction: multi-scan (*SADABS*; Bruker, 2000)	*h* = −14→15
*T*_min_ = 0.368, *T*_max_ = 0.753	*k* = −12→12
13742 measured reflections	*l* = −17→17

### Refinement

**Table:**

Refinement on *F*^2^	Primary atom site location: structure-invariant direct methods
Least-squares matrix: full	Secondary atom site location: difference Fourier map
*R*[*F*^2^ > 2σ(*F*^2^)] = 0.030	Hydrogen site location: inferred from neighbouring sites
*wR*(*F*^2^) = 0.079	H-atom parameters constrained
*S* = 1.06	*w* = 1/[σ^2^(*F*_o_^2^) + (0.0456*P*)^2^ + 0.6227*P*] where *P* = (*F*_o_^2^ + 2*F*_c_^2^)/3
3575 reflections	(Δ/σ)_max_ = 0.002
235 parameters	Δρ_max_ = 0.67 e Å^−^^3^
0 restraints	Δρ_min_ = −0.17 e Å^−^^3^

### Special details

**Table:**

Geometry. Bond distances, angles *etc*. have been calculated using the rounded fractional coordinates. All su's are estimated from the variances of the (full) variance-covariance matrix. The cell e.s.d.'s are taken into account in the estimation of distances, angles and torsion angles
Refinement. Refinement on *F*^2^ for ALL reflections except those flagged by the user for potential systematic errors. Weighted *R*-factors *wR* and all goodnesses of fit *S* are based on *F*^2^, conventional *R*-factors *R* are based on *F*, with *F* set to zero for negative *F*^2^. The observed criterion of *F*^2^ > σ(*F*^2^) is used only for calculating -*R*-factor-obs *etc*. and is not relevant to the choice of reflections for refinement. *R*-factors based on *F*^2^ are statistically about twice as large as those based on *F*, and *R*-factors based on ALL data will be even larger.

### Fractional atomic coordinates and isotropic or equivalent isotropic displacement parameters (Å^2^)

**Table:**

	*x*	*y*	*z*	*U*_iso_*/*U*_eq_	
Br1	0.23271 (2)	1.04855 (2)	0.59316 (1)	0.0359 (1)	
O1	0.13462 (11)	0.13416 (13)	0.33354 (11)	0.0333 (4)	
O2	−0.09408 (11)	0.40741 (15)	0.32404 (10)	0.0329 (4)	
N1	0.13907 (13)	0.45872 (14)	0.37523 (11)	0.0244 (5)	
N2	0.07047 (14)	0.55054 (14)	0.39738 (12)	0.0259 (5)	
C1	0.11110 (16)	0.66570 (18)	0.44103 (13)	0.0244 (5)	
C2	0.03445 (16)	0.74538 (19)	0.47975 (14)	0.0275 (6)	
C3	0.07041 (16)	0.85857 (19)	0.52624 (14)	0.0283 (6)	
C4	0.18176 (17)	0.89302 (18)	0.53100 (13)	0.0272 (6)	
C5	0.25851 (16)	0.81515 (19)	0.49182 (14)	0.0294 (6)	
C6	0.22285 (16)	0.70074 (19)	0.44637 (13)	0.0280 (6)	
C7	0.29704 (16)	0.25718 (18)	0.31885 (13)	0.0255 (5)	
C8	0.34273 (16)	0.3676 (2)	0.28233 (14)	0.0294 (6)	
C9	0.45584 (17)	0.3718 (2)	0.27136 (16)	0.0358 (7)	
C10	0.52336 (18)	0.2658 (2)	0.29719 (16)	0.0402 (7)	
C11	0.47871 (18)	0.1550 (2)	0.33354 (16)	0.0370 (7)	
C12	0.36607 (17)	0.1503 (2)	0.34456 (14)	0.0304 (6)	
C13	−0.05567 (15)	0.24899 (18)	0.21378 (13)	0.0241 (5)	
C14	0.00727 (16)	0.23913 (18)	0.13846 (14)	0.0277 (6)	
C15	−0.03124 (18)	0.1653 (2)	0.05974 (14)	0.0332 (6)	
C16	−0.13105 (19)	0.0989 (2)	0.05795 (16)	0.0369 (7)	
C17	−0.19298 (18)	0.1058 (2)	0.13385 (17)	0.0373 (7)	
C18	−0.15682 (16)	0.1820 (2)	0.21102 (15)	0.0306 (6)	
C19	0.17494 (16)	0.24228 (18)	0.32685 (13)	0.0250 (6)	
C20	0.09791 (15)	0.35573 (18)	0.32905 (13)	0.0245 (5)	
C21	−0.02208 (15)	0.33948 (18)	0.29325 (13)	0.0251 (5)	
H2	−0.04240	0.72210	0.47430	0.0330*	
H3	0.01900	0.91180	0.55450	0.0340*	
H5	0.33500	0.83990	0.49600	0.0350*	
H6	0.27470	0.64690	0.41910	0.0340*	
H8	0.29640	0.44040	0.26480	0.0350*	
H9	0.48690	0.44720	0.24620	0.0430*	
H10	0.60090	0.26910	0.28990	0.0480*	
H11	0.52540	0.08250	0.35080	0.0440*	
H12	0.33530	0.07460	0.36960	0.0360*	
H14	0.07700	0.28290	0.14060	0.0330*	
H15	0.01100	0.16070	0.00740	0.0400*	
H16	−0.15730	0.04810	0.00440	0.0440*	
H17	−0.26070	0.05800	0.13290	0.0450*	
H18	−0.20080	0.18880	0.26210	0.0370*	
H21	−0.00220	0.53940	0.38450	0.0310*	

### Atomic displacement parameters (Å^2^)

**Table:**

	*U*^11^	*U*^22^	*U*^33^	*U*^12^	*U*^13^	*U*^23^
Br1	0.0364 (1)	0.0287 (1)	0.0424 (1)	−0.0006 (1)	0.0032 (1)	−0.0091 (1)
O1	0.0286 (7)	0.0262 (7)	0.0452 (8)	0.0008 (6)	0.0049 (6)	0.0005 (6)
O2	0.0243 (7)	0.0386 (8)	0.0362 (8)	0.0060 (6)	0.0050 (6)	−0.0053 (7)
N1	0.0248 (8)	0.0254 (8)	0.0234 (8)	0.0059 (6)	0.0041 (6)	0.0006 (6)
N2	0.0227 (8)	0.0264 (8)	0.0285 (8)	0.0039 (6)	0.0027 (7)	−0.0031 (6)
C1	0.0285 (10)	0.0237 (9)	0.0207 (8)	0.0033 (7)	0.0019 (7)	0.0018 (7)
C2	0.0249 (10)	0.0280 (10)	0.0302 (10)	0.0026 (7)	0.0053 (8)	0.0007 (8)
C3	0.0301 (10)	0.0260 (9)	0.0297 (10)	0.0063 (8)	0.0076 (8)	−0.0003 (8)
C4	0.0334 (11)	0.0233 (9)	0.0250 (9)	0.0013 (8)	0.0038 (8)	−0.0012 (8)
C5	0.0240 (10)	0.0312 (10)	0.0332 (10)	0.0012 (8)	0.0036 (8)	0.0003 (8)
C6	0.0267 (10)	0.0284 (10)	0.0293 (10)	0.0067 (8)	0.0056 (8)	−0.0007 (8)
C7	0.0247 (9)	0.0277 (10)	0.0233 (9)	0.0026 (7)	−0.0001 (7)	−0.0049 (7)
C8	0.0270 (10)	0.0314 (10)	0.0297 (10)	0.0023 (8)	0.0027 (8)	−0.0021 (8)
C9	0.0290 (11)	0.0394 (12)	0.0396 (12)	−0.0045 (9)	0.0060 (9)	−0.0046 (9)
C10	0.0235 (10)	0.0536 (14)	0.0435 (13)	0.0021 (10)	0.0038 (9)	−0.0133 (11)
C11	0.0292 (11)	0.0385 (12)	0.0416 (12)	0.0121 (9)	−0.0034 (9)	−0.0095 (10)
C12	0.0293 (10)	0.0299 (10)	0.0308 (10)	0.0052 (8)	−0.0013 (8)	−0.0056 (8)
C13	0.0202 (9)	0.0232 (9)	0.0284 (9)	0.0030 (7)	0.0008 (7)	0.0041 (7)
C14	0.0227 (9)	0.0289 (10)	0.0314 (10)	−0.0029 (8)	0.0030 (8)	0.0013 (8)
C15	0.0368 (11)	0.0349 (11)	0.0279 (10)	0.0005 (9)	0.0035 (8)	−0.0016 (8)
C16	0.0394 (12)	0.0319 (11)	0.0363 (11)	−0.0022 (9)	−0.0096 (10)	−0.0028 (9)
C17	0.0260 (10)	0.0335 (11)	0.0502 (13)	−0.0074 (8)	−0.0051 (9)	0.0049 (10)
C18	0.0236 (9)	0.0324 (11)	0.0359 (11)	−0.0002 (8)	0.0040 (8)	0.0074 (9)
C19	0.0255 (10)	0.0250 (10)	0.0241 (9)	0.0021 (7)	0.0005 (7)	−0.0017 (7)
C20	0.0238 (9)	0.0256 (9)	0.0239 (9)	0.0033 (7)	0.0025 (7)	−0.0002 (7)
C21	0.0240 (9)	0.0239 (9)	0.0277 (9)	0.0024 (7)	0.0049 (7)	0.0035 (8)

### Geometric parameters (Å, °)

**Table:**

Br1—C4	1.8985 (19)	C13—C21	1.488 (3)
O1—C19	1.222 (2)	C14—C15	1.392 (3)
O2—C21	1.232 (2)	C15—C16	1.379 (3)
N1—N2	1.317 (2)	C16—C17	1.383 (3)
N1—C20	1.315 (2)	C17—C18	1.382 (3)
N2—C1	1.402 (2)	C19—C20	1.494 (3)
N2—H21	0.8800	C20—C21	1.485 (3)
C1—C6	1.385 (3)	C2—H2	0.9500
C1—C2	1.394 (3)	C3—H3	0.9500
C2—C3	1.386 (3)	C5—H5	0.9500
C3—C4	1.380 (3)	C6—H6	0.9500
C4—C5	1.387 (3)	C8—H8	0.9500
C5—C6	1.389 (3)	C9—H9	0.9500
C7—C8	1.389 (3)	C10—H10	0.9500
C7—C19	1.494 (3)	C11—H11	0.9500
C7—C12	1.402 (3)	C12—H12	0.9500
C8—C9	1.387 (3)	C14—H14	0.9500
C9—C10	1.385 (3)	C15—H15	0.9500
C10—C11	1.386 (3)	C16—H16	0.9500
C11—C12	1.382 (3)	C17—H17	0.9500
C13—C14	1.386 (3)	C18—H18	0.9500
C13—C18	1.395 (3)		
			
Br1···C17^i^	3.700 (2)	C4···H18^iii^	3.0500
Br1···C18^i^	3.433 (2)	C5···H14^viii^	2.9000
Br1···C13^i^	3.5778 (19)	C6···H16^vi^	3.0100
Br1···H11^ii^	3.2300	C6···H14^viii^	2.9500
Br1···H18^iii^	3.2500	C14···H6^iv^	2.9900
O1···C13	2.945 (2)	C14···H5^iv^	3.0300
O1···C14	3.209 (2)	C15···H6^iv^	3.0700
O1···C8^iv^	3.231 (2)	C19···H14	2.8100
O1···C9^iv^	3.218 (3)	C20···H14	2.7700
O1···C3^iii^	3.346 (2)	C20···H8	2.7900
O2···N1	2.866 (2)	C20···H2^iii^	3.0600
O2···C2^iii^	3.220 (2)	C21···H21	2.4300
O2···C17^v^	3.382 (3)	H2···H21	2.3600
O2···N2	2.592 (2)	H2···C20^iii^	3.0600
O1···H8^iv^	2.6300	H3···O1^iii^	2.6200
O1···H12	2.4900	H3···C3^vii^	2.7900
O1···H3^iii^	2.6200	H3···H3^vii^	2.4000
O1···H9^iv^	2.6000	H5···C14^viii^	3.0300
O2···H18	2.6900	H5···H14^viii^	2.3900
O2···H17^v^	2.4600	H6···N1	2.5600
O2···H21	1.9000	H6···C14^viii^	2.9900
N1···O2	2.866 (2)	H6···C15^viii^	3.0700
N1···C8	3.060 (3)	H6···H14^viii^	2.4900
N2···O2	2.592 (2)	H6···H16^vi^	2.4400
N1···H8	2.6000	H8···N1	2.6000
N1···H16^vi^	2.9000	H8···C20	2.7900
N1···H6	2.5600	H8···O1^viii^	2.6300
C2···O2^iii^	3.220 (2)	H9···O1^viii^	2.6000
C2···C20^iii^	3.469 (3)	H10···H18^xi^	2.6000
C2···C21^iii^	3.373 (3)	H11···Br1^ii^	3.2300
C3···O1^iii^	3.346 (2)	H12···O1	2.4900
C3···C3^vii^	3.410 (3)	H14···C19	2.8100
C3···C21^iii^	3.386 (3)	H14···C20	2.7700
C8···O1^viii^	3.231 (2)	H14···C5^iv^	2.9000
C8···N1	3.060 (3)	H14···C6^iv^	2.9500
C9···O1^viii^	3.218 (3)	H14···H5^iv^	2.3900
C13···O1	2.945 (2)	H14···H6^iv^	2.4900
C13···Br1^ix^	3.5778 (19)	H16···N1^xii^	2.9000
C14···C19	3.173 (3)	H16···C6^xii^	3.0100
C14···O1	3.209 (2)	H16···H6^xii^	2.4400
C17···O2^x^	3.382 (3)	H17···O2^x^	2.4600
C17···Br1^ix^	3.700 (2)	H18···O2	2.6900
C18···Br1^ix^	3.433 (2)	H18···H10^xiii^	2.6000
C19···C14	3.173 (3)	H18···Br1^iii^	3.2500
C20···C2^iii^	3.469 (3)	H18···C4^iii^	3.0500
C21···C2^iii^	3.373 (3)	H21···O2	1.9000
C21···C3^iii^	3.386 (3)	H21···C21	2.4300
C3···H3^vii^	2.7900	H21···H2	2.3600
			
N2—N1—C20	119.24 (16)	C19—C20—C21	119.06 (16)
N1—N2—C1	121.18 (16)	N1—C20—C19	116.22 (16)
N1—N2—H21	119.00	C13—C21—C20	120.18 (16)
C1—N2—H21	119.00	O2—C21—C13	119.06 (16)
N2—C1—C2	117.45 (17)	O2—C21—C20	120.55 (17)
N2—C1—C6	122.17 (17)	C1—C2—H2	120.00
C2—C1—C6	120.38 (17)	C3—C2—H2	120.00
C1—C2—C3	120.00 (18)	C2—C3—H3	120.00
C2—C3—C4	119.26 (18)	C4—C3—H3	120.00
C3—C4—C5	121.19 (18)	C4—C5—H5	120.00
Br1—C4—C3	120.03 (15)	C6—C5—H5	120.00
Br1—C4—C5	118.78 (15)	C1—C6—H6	120.00
C4—C5—C6	119.59 (18)	C5—C6—H6	120.00
C1—C6—C5	119.56 (18)	C7—C8—H8	120.00
C12—C7—C19	117.24 (17)	C9—C8—H8	120.00
C8—C7—C12	119.53 (18)	C8—C9—H9	120.00
C8—C7—C19	123.12 (17)	C10—C9—H9	120.00
C7—C8—C9	120.13 (19)	C9—C10—H10	120.00
C8—C9—C10	119.89 (19)	C11—C10—H10	120.00
C9—C10—C11	120.5 (2)	C10—C11—H11	120.00
C10—C11—C12	119.8 (2)	C12—C11—H11	120.00
C7—C12—C11	120.12 (19)	C7—C12—H12	120.00
C14—C13—C21	120.75 (17)	C11—C12—H12	120.00
C14—C13—C18	119.50 (18)	C13—C14—H14	120.00
C18—C13—C21	119.50 (17)	C15—C14—H14	120.00
C13—C14—C15	120.28 (18)	C14—C15—H15	120.00
C14—C15—C16	119.73 (19)	C16—C15—H15	120.00
C15—C16—C17	120.3 (2)	C15—C16—H16	120.00
C16—C17—C18	120.3 (2)	C17—C16—H16	120.00
C13—C18—C17	119.92 (19)	C16—C17—H17	120.00
O1—C19—C20	117.28 (17)	C18—C17—H17	120.00
O1—C19—C7	120.11 (17)	C13—C18—H18	120.00
C7—C19—C20	122.60 (16)	C17—C18—H18	120.00
N1—C20—C21	124.02 (17)		
			
C20—N1—N2—C1	−175.78 (17)	C8—C9—C10—C11	0.3 (3)
N2—N1—C20—C21	4.7 (3)	C9—C10—C11—C12	−0.3 (3)
N2—N1—C20—C19	−165.54 (16)	C10—C11—C12—C7	0.2 (3)
N1—N2—C1—C6	13.0 (3)	C18—C13—C14—C15	1.4 (3)
N1—N2—C1—C2	−166.92 (17)	C21—C13—C18—C17	174.85 (18)
N2—C1—C6—C5	−179.01 (18)	C14—C13—C21—O2	135.6 (2)
C2—C1—C6—C5	0.9 (3)	C14—C13—C21—C20	−39.1 (3)
N2—C1—C2—C3	178.10 (18)	C18—C13—C21—O2	−38.7 (3)
C6—C1—C2—C3	−1.8 (3)	C18—C13—C21—C20	146.63 (18)
C1—C2—C3—C4	2.0 (3)	C21—C13—C14—C15	−172.87 (18)
C2—C3—C4—C5	−1.3 (3)	C14—C13—C18—C17	0.5 (3)
C2—C3—C4—Br1	179.34 (15)	C13—C14—C15—C16	−1.8 (3)
C3—C4—C5—C6	0.4 (3)	C14—C15—C16—C17	0.2 (3)
Br1—C4—C5—C6	179.76 (14)	C15—C16—C17—C18	1.7 (3)
C4—C5—C6—C1	−0.2 (3)	C16—C17—C18—C13	−2.1 (3)
C19—C7—C8—C9	−175.90 (19)	O1—C19—C20—N1	141.39 (18)
C8—C7—C12—C11	0.0 (3)	C7—C19—C20—C21	151.86 (17)
C8—C7—C19—O1	159.27 (19)	O1—C19—C20—C21	−29.4 (3)
C8—C7—C19—C20	−22.0 (3)	C7—C19—C20—N1	−37.4 (3)
C19—C7—C12—C11	176.17 (18)	N1—C20—C21—O2	−17.5 (3)
C12—C7—C8—C9	0.1 (3)	N1—C20—C21—C13	157.07 (18)
C12—C7—C19—C20	161.96 (18)	C19—C20—C21—O2	152.44 (18)
C12—C7—C19—O1	−16.8 (3)	C19—C20—C21—C13	−33.0 (3)
C7—C8—C9—C10	−0.2 (3)		

Symmetry codes: (i) *x*+1/2, −*y*+3/2, *z*+1/2; (ii) −*x*+1, −*y*+1, −*z*+1; (iii) −*x*, −*y*+1, −*z*+1; (iv) −*x*+1/2, *y*−1/2, −*z*+1/2; (v) −*x*−1/2, *y*+1/2, −*z*+1/2; (vi) *x*+1/2, −*y*+1/2, *z*+1/2; (vii) −*x*, −*y*+2, −*z*+1; (viii) −*x*+1/2, *y*+1/2, −*z*+1/2; (ix) *x*−1/2, −*y*+3/2, *z*−1/2; (x) −*x*−1/2, *y*−1/2, −*z*+1/2; (xi) *x*+1, *y*, *z*; (xii) *x*−1/2, −*y*+1/2, *z*−1/2; (xiii) *x*−1, *y*, *z*.

### Hydrogen-bond geometry (Å, °)

**Table:**

*D*—H···*A*	*D*—H	H···*A*	*D*···*A*	*D*—H···*A*
N2—H21···O2	0.88	1.90	2.592 (2)	135
C8—H8···N1	0.95	2.60	3.060 (3)	110
C17—H17···O2^x^	0.95	2.46	3.382 (3)	162

Symmetry codes: (x) −*x*−1/2, *y*−1/2, −*z*+1/2.
